# Cytotoxicity of Protein-Carbon Nanotubes on J774 Macrophages Is a Functionalization Grade-Dependent Effect

**DOI:** 10.1155/2015/796456

**Published:** 2015-05-17

**Authors:** Silvia Lorena Montes-Fonseca, Blanca Sánchez-Ramírez, Antonia Luna-Velasco, Carlos Arzate-Quintana, Macrina Beatriz Silva-Cazares, Carmen González Horta, Erasmo Orrantia-Borunda

**Affiliations:** ^1^Centro de Investigación en Materiales Avanzados (CIMAV), Miguel de Cervantes 120, Complejo Industrial Chihuahua, 31109 Chihuahua, CHIH, Mexico; ^2^Facultad de Ciencias Químicas, Universidad Autónoma de Chihuahua, Nuevo Campus Universitario, Circuito No. 1, 31125 Chihuahua, CHIH, Mexico; ^3^Coordinación Académica Región Altiplano, Universidad Autónoma de San Luis Potosí, 78000 Matehuala, SLP, Mexico

## Abstract

Carbon nanotubes (CNTs) are used as carriers in medicine due to their ability to be functionalized with chemical substances. However, cytotoxicity analysis is required prior to use for *in vivo* models. The aim of this study was to evaluate the cytotoxic effect of CNTs functionalized with a 46 kDa surface protein from *Entamoeba histolytica* (P46-CNTs) on J774A macrophages. With this purpose, CNTs were synthesized by spray pyrolysis and purified (P-CNTs) using sonication for 48 h. A 46 kDa protein, with a 4.6–5.4 pI range, was isolated from *E. histolytica* HM1:IMSS strain trophozoites using an OFFGEL system. The P-CNTs were functionalized with the purified 46 kDa protein, classified according to their degree of functionalization, and characterized by Raman and Infrared spectroscopy. *In vitro* cytotoxicity was evaluated by MTT, apoptosis, and morphological assays. The results demonstrated that P46-CNTs exhibited cytotoxicity dependent upon the functionalized grade. Contrary to what was expected, P46-CNTs with a high grade of functionalization were more toxic to J774 macrophages than P46-CNTs with a low grade of functionalization, than P-CNTs, and had a similar level of toxicity as UP-CNT. This suggests that the nature of the functionalized protein plays a key role in the cytotoxicity of these nanoparticles.

## 1. Introduction

Carbon nanotubes (CNTs) are carbon allotropes with a size range of <100 nm. These nanoparticles are used in nanomedicine as carrier systems of drugs due their ability of functionalization. Nanoparticles offer better pharmacokinetic properties, such as controlled and sustained release and targeting of specific cells, tissues, or organs [1]. Consequently CNTs have been employed to deliver drugs, genes, vaccines, and diagnostics [2]. However, results obtained in toxicity studies of CNTs are contradictory. These contradictions undoubtedly arise as a result of variations in synthesis and preparation methods used [3]. These variations are due to different features of the CNTs that depend on its physicochemical modifications. Montes-Fonseca et al. (2012) proved that purified CNTs (P-CNTs) that had a length of <1 *μ*m and 7% of COOH groups on its surface had a minor toxic effect compared to larger P-CNTs containing a minor percentage of groups COOH [4]. The COOH groups increase both solubility and stability of CNTs in aqueous solution due to their charges [5]. Another study demonstrated that functionalized CNTs (*f*-CNTs) with different molecules showed a decrease in its cytotoxic effect, which could be attributed to a higher compatibility of* f-*CNTs with the cell membrane [6]. However, some studies have demonstrated that functionalization does not totally eliminate the toxicity of* f-*CNTs because this process improves the biodistribution of the molecules attached. Therefore, the functionalization may potentiate the natural toxic effect of a substance [6].

Thus it is important to assess the toxicity of* f*-CNTs, especially those that could be used in a biologic system, such as nanovaccines. Lastly, peptides capable of eliciting an immune response against a pathogen of interest are selected and functionalized on CNTs [7]. A particular case of our interest is amoebiasis, an intestinal infection caused by the parasite* Entamoeba histolytica*, which afflicts several million people worldwide [8]. Transmission occurs via the fecal-oral route, either directly by person-to-person contact or indirectly by eating or drinking fecal contaminated food or water. Although infection can occur at any age, a higher prevalence has been observed in school-age populations with lower socioeconomic levels [9]. In Mexico, the prevalence is estimated at 4 to 20% in some populations [10]. To date, a vaccine does not exist that evokes a specific and effective immune response against this parasite.

The aim of this study was to evaluate the cytotoxicity of* f*-CNTs with an* Entamoeba histolytica* surface protein with different functionalization degrees on J774A macrophages.

## 2. Methods

### 2.1. Synthesis and Purification of CNTs

CNTs were synthesized by spray pyrolysis, using toluene and ferrocene as the carbon source and the catalyst, respectively [11]. The synthesis time was 2 min, purification was carried out with 0.2 g of crude unpurified CNTs (UP-CNTs) suspended in 400 mL of a mixture of concentrated H_2_SO_4_ (90%)/HNO_3_ (70%) 3 : 1 v/v, and particles were sonicated in a water bath for 48 h [12]. The resultant purified CNTs (P-CNTs) were collected by filtration through a 450 nm pore size polytetrafluoroethylene filter and washed four times with water and methanol. Finally, P-CNTs were dried at room temperature [13].

### 2.2. Isolation of* Entamoeba histolytica* Surface Protein of 46 kDa

Amoebic protein was obtained from an axenic culture of* Entamoeba histolytica* HM-1:IMSS. Briefly, trophozoites were harvested by centrifugation and resuspended in buffer A (0.05 M Tris-HCl pH 6.8, added with 5% Triton X-100 and a protease inhibitor cocktail containing 1 mM phenylmethylsulfonylfluoride (Sigma, Chemical Co.), 2 *μ*M leupeptin, and 5 mM N-ethylmaleimide (Sigma, Chemical Co.)). The trophozoites were lysed in a Teflon glass Potter homogenizer coupled to a drill at 3000 rpm with 80 up and down strokes in an ice bath. Homogenate was centrifuged for 30 min at 12,000 rpm at 4°C and the obtained pellet was suspended in 0.36 mL of buffer A and 1.44 mL of OFFGEL buffer stock (1.25X) (Agilent Technologies, St. Clara, USA). Later, proteins were separated according to their isoelectric point (pI) using the Agilent 3100 OFFGEL fractionator (Agilent Technologies, St. Clara, USA), pH gradient ranging from 3 to 10. One aliquot of each batch, was screening in 2100 BioAnalyzer (Agilent Technologies, St. Clara, USA) to identify a single protein fraction; finally, the fraction corresponding to pH 4.6–5.4, which contained a 46 kDa protein, was cleaned using the ReadyPrep-2D CleanUp kit (BioRad) to eliminate salts and buffers that could interfere in the functionalization procedure. The protein was quantified using the Bradford assay [14] and stored at −70°C until use.

### 2.3. Functionalization of P-CNTs

The P-CNTs were functionalized with the 46 kDa protein by diimide-activated amidation according to the methodology described by Huang et al. (2002) [15] with modifications as described: 5.5 mg of P-CNTs and 10 mg of 1-ethyl-3-(3-dimethylaminopropyl) carbodiimide were added to 10 mL of 0.1 M phosphate buffer pH 7.5. The suspension was sonicated for 2 h and 66.74 *μ*g of the 46 kDa protein was added and mixed for 24 h at 4°C. Next, the suspension was centrifuged for 30 min at 5,000 rpm, to separate* f*-CNTs according to their solubility. The* f-*CNTs recovered from the supernatant and precipitate were designated as P46S-CNTs and P46P-CNTs, respectively. Both, P46S-CNT and P46P-CNT, were filtered separately through a 0.45 *μ*m pore size membrane filter. Finally,* f-*CNTs were suspended in 2 mL of deionized water and lyophilized to dryness in a FreeZone Triad Freeze Dry System (Labconco Co., Kansas, USA).

### 2.4. Characterization of* f-*CNTs

The* f*-CNTs were characterized by Raman and Infrared spectroscopy using micro-Raman LabRAM HR (Horiba Jobn Yvon, NJ, USA) coupled to an Olympus BX-4 microscope and Spectrum Gx (Perkin Elmer, Massachusetts, USA).

### 2.5. Viability Test in J774 Macrophage (MOs) Cell Line

Cell viability was determined by MTT assays (Sigma-Aldrich) in 96-well plates. For this assay, 10^5^ cells were cultivated in DMEM-HG supplemented with 10% heat-inactivated bovine fetal serum, 100 IU/mL of penicillin, 100 *μ*m/mL streptomycin, and 2 mM L-glutamine; cells were interacted with UP-CNTs, P-CNTs, or P46P-CNTs at concentrations of 0.06, 0.6, and 6 mg/L. Due to the low production yield, P46S-CNTs were tested only at a single concentration. Cultures were incubated for 24 h at 37°C in a humid atmosphere at 5% CO_2_. MOs without stimulus were used as control. At 20 h cultivation time, 0.1 mg of MTT dissolved in sterile phosphate buffered saline was added to each well, followed by incubation for 4 or more hours. Cellular lysis was conducted with acidified isopropanol, and the absorbance at 590 nm was quantified using a Varioskan Flash microreader (Thermo Scientific, Waltham, MA, USA).

### 2.6. Apoptosis Assay

The apoptosis assays were performed using the ReadiPlate 96 EnzChek Caspase-3 Assay Kit (Molecular Probes, Waltham, MA, USA). For this, 10^6^ MOs were cultured in 24-well plates using the same conditions as the MTT assay. MOs were lysed in 1X buffer provided by the caspase-3 kit for 30 min in an ice bath (4°C). The lysate was centrifuged for 5 min at 5,000 rpm, and 50 *μ*L of each supernatant was incubated in the plates provided by the kit. Fluorescence intensity was determined at 496/520 nm Ex/Em using a Varioskan Flash microreader (Thermo Scientific, Waltham, MA, USA).

### 2.7. Morphological Analysis

Exposures were performed in the Lab Tek Chamber slide system (Nalge Nunc International, Rochester, NY, USA) using 10^6^ cells/100 *μ*L per well incubated with 6 mg/L of the different CNTs for 24 h. Next, the supernatant was removed and the cells were fixed and stained using fast Hemostain (Hycel, DF, Mexico). Photomicrographs were obtained using an Olympus BX4-1 microscope (Olympus, Miami, Florida, USA) equipped with a Pixera cold-coupled device camera (Pixera, Miami, Florida, USA) and analyzed with IMAGE Pro-Plus 4.1 software (Media Cybernetics, Silver Spring, Maryland, USA).

### 2.8. Statistical Analysis

Statistical analysis was carried out using Minitab software (State College, Pennsylvania); a one-way analysis of variance was performed to determine the difference between the MOs interactions with different CNTs at the concentrations tested.

## 3. Results and Discussion

### 3.1. Characterization of* f*-CNTs

The UP-CNTs and P-CNTs used were previously characterized by our research group [4]. UP-CNTs were on average 20–40 nm in diameter and 30 *μ*m in length. P-CNTs obtained by sonication with H_2_SO_4_/HNO_3_ 3 : 1 v/v showed a considerable decrease in length to <1 *μ*m and an increase to 7% of COOH groups.

Regarding the functionalization of P-CNTs with the 46 kDa* Entamoeba histolytica* surface protein, in this study a centrifugation step was implemented at the end of the functionalization process to separate* f*-CNTs according to their solubility, where* f*-CNTs with a high grade of functionalization, which were more soluble, remained in the supernatant and were named P46S-CNTs [15]. To assess the functionalization degree of the P46-CNTs recovered from supernatant or precipitate (P46P-CNTs), both* f*-CNTs were characterized by Raman and Infrared spectroscopy.

**Figure 1:**
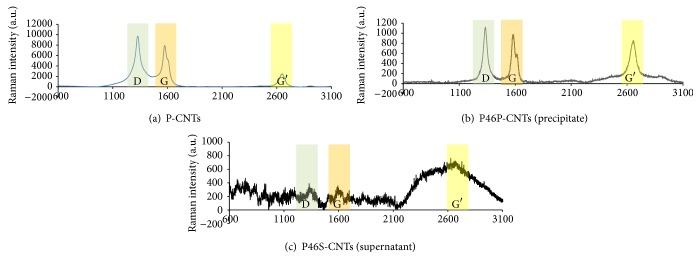
Raman spectra of different CNTs. Raman spectra obtained from P-CNTs (a), P46P-CNTs (b), and P46S-CNTs (c). Each figure shows the D band at 1330 cm^−1^, the G-band at 1600 cm^−1^, and the G′ band at 2650 cm^−1^ (laser excitation 632.8 nm).

The Raman analysis spectra obtained for P-CNTs (Figure 1(a)), P46P-CNTs (Figure 1(b)), and P46S-CNTs (Figure 1(c)) displayed D, G, and G′ bands at 1330 cm^−1^, 1600 cm^−1^, and 2650 cm^−1^, respectively; however, differences in intensity were observed. Patterns of intensity in the D and G bands were similar between P-CNTs and P46P-CNTs, while in P46S-CNTs the intensity of these bands decreased considerably. In contrast, the G′ band displayed a higher intensity in both P46P-CNTs and P46S-CNTs, compared with P-CNTs. In addition, Raman spectra of P46S-CNTs had numerous unspecific bands along the spectra.

The D, G, and G′ bands correspond to different vibrations of the carbon atoms on the graphitic layers, and their intensities depend on the structural quality of the CNTs [16]. In particular the G′ band corresponds to an overtone of the D band and its intensity increases when CNTs are submitted to mechanical stresses such as stretching and compression [16]. These results suggest that the functionalization and lyophilization processes induce a mechanical stress independently of their functionalization degree, thus explaining the increase in the G′ band intensity for both functionalized particles. On the other hand, a decrease of the D and G band intensities coupled with the disorder introduced the sp2 carbon network in the P46S-CNTs spectra, which suggests the presence of functional groups attached to the surface of CNTs [17]. However, this type of analysis does not allow us to determine the composition of these functional groups.

**Figure 2:**
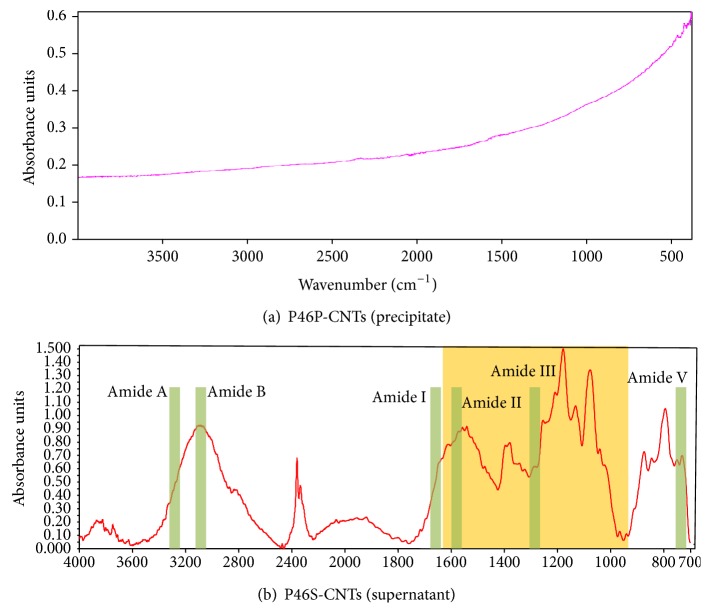
Infrared spectra of different CNTs. Infrared spectra obtained from P46P-CNTs (a) and P46S-CNTs (b) where the green boxes show the 6 principal amide-bands at 3300 cm^−1^ (Amide A), 3100 cm^−1^ (Amide B), 1650 cm^−1^ (Amide I), 1550 cm^−1^ (Amide II), 1300 cm^−1^ (Amide III), and 725 cm^−1^ (Amide V); the yellow box represents amino acid specific signals at 1630–1000 cm^−1^.

Infrared (IR) spectroscopy studies, on the other hand, are used to identify the functional groups added to the walls of CNTs. However, the activity associated with these groups is frequently difficult to observe [17]. Therefore, it is preferable to have at least 5% wt of the functionalized groups on the sample to detect IR activity. If percentage is lower, the optical absorption spectrum, will be dominated by the electronic contribution of the CNTs and not by the functionalized groups. The IR spectra of P46P-CNTs (Figure 2(a)) contained two weak peaks at 868 cm^−1^ and 1588 cm^−1^, which are characteristic of CNTs [18]. Signals due to functionalized groups were not detected in these particles. For the case of P46S-CNTs, the IR spectra presented multiple peaks as a result of functional groups attached to the CNTs, which were detected at different wavelengths (Figure 2(b)).

According to Arrondo et al. (1993), the IR spectra of the protein are composed of nine specific bands, which correspond to the different vibrations of the peptide bond (CONH), called amide bands [19]. In Figure 2(b), green squares indicate the wavelengths of the main amide bands as well as their corresponding spectra signal. Moreover, we also observed multiple peaks at lengths of 1000–1630 cm^−1^, which according to Barth (2000) correspond to specific vibration bands of different amino acids [20]. Also in the spectra, a broad band at 3000 to 2850 cm^−1^ that corresponds to the carbohydrate-OH group's vibration was detected. Taken together these results suggest that the substance attached to the CNTs-P46S could be a glycoprotein. Furthermore, we may conclude that the centrifugation step at the end of the functionalization process enabled us to effectively separate the* f-*CNTs according to the density of the chemical groups attached to their surface. Although P46P-CNTs showed no signs that indicate the presence of functional groups, we must remember that the techniques used are insensitive and may not detect substances at concentrations less than 5% wt. In contrast, for the P46S-CNTs, besides having a higher aqueous stability, the Raman and IR spectra indicate the presence of an increased amount of attached functional groups.

### 3.2. Cytotoxic Assays

**Figure 3:**
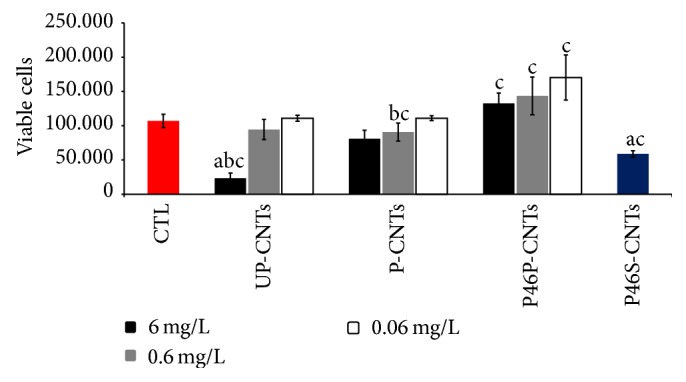
Viability of MOs exposed to different CNTs for 24 h. Each bar represents mean ± SD of two experiments in triplicate (*n* = 6). a, *P* < 0.01 denotes significant differences between mean values measured in the indicated group compared to the control without stimulus (CTL); b, *P* < 0.01 denotes significant differences between mean values for CNTs at different concentrations; c, *P* < 0.01 denotes significant differences between mean values for a particular concentration among different CNTs.

Cytotoxicity of different CNTs was assessed by MTT assays on J774 MOs cultures; results are shown in Figure 3. As previously reported by our research group [4], MOs exposed to 6 mg/L UP-CNTs had a greater toxic effect compared to other nanoparticles, observing an 80% decrease in cell viability. At concentrations of 0.6 and 0.06 mg/L no difference in cell viability was found compared with the control without stimulus. MOs exposed to P-CNTs resulted in a dose-dependent decrease in cell viability; however no significant difference with control, at any concentration tested, was detected.

Previous studies have demonstrated that UP-CNTs had toxicity at high concentrations independently of their size in different cell lines [4, 12, 21, 22]. Nevertheless, at concentrations lower than 1 mg/L, UP-CNTs toxicity is dependent on size, where CNTs with lengths greater than 100 *μ*m produce higher toxic effects compared to CNTs with lengths shorter than 30 *μ*m in cell cultures [4, 12, 21–23]. The results found in this study agree with the previous studies. Purification is a process used to eliminate catalyst wastes such as Fe and Ni deposited on the surface of UP-CNTs during its synthesis besides conferring greater stability in aqueous solution due to the oxidized groups attached (COOH, OH, and CO) [13]. Our research group found less toxic effects in MOs exposed to P-CNTs, which has already been reported [4]. However, other studies have reported a higher toxic effect by P-CNTs compared with UP-CNTs [24, 25]. This difference most likely depends on chemical and structural modifications originating from the purification procedure. P-CNTs used in this study had a length < 1 *μ*m, as well as 98.2% purity and 7% COOH groups. P-CNTs with a smaller size and a higher percentage of COOH groups exhibit less toxic effects due to its high degree of dispersion in an aqueous solution [26].

Dispersion of CNTs can also be obtained by functionalization. Numerous studies have demonstrated that* f*-CNTs have less toxic effects compared to UP-CNTs and P-CNTs [27].

On the other hand, MOs exposed to P46P-CNTs did an increase in cell viability by 30% at 0.06 mg/L, which was not significant when compared with control or with P-CNTs at the same concentration. Finally, MOs exposed to P46S-CNTs resulted in a significant decrease in cell viability by 50% compared to control. Earlier studies of cell viability in MOs exposed to* f-*CNTs with a protein of 220 kDa from* Entamoeba histolytica *[12] obtained results similar to those found in this study with P46P-CNTs, in which cell proliferation observed with this nanoparticle at a concentration of 0.06 mg/L was most likely due to the recognition of the functionalized protein by the MOs; however, additional studies are needed. Furthermore, the P46S-CNTs resulted in contradictory behavior because, contrary to expectations, these nanoparticles were more toxic than P46P-CNTs. Other investigators have also observed that some* f-*CNTs can induce toxicity and that the response depends on both the protein or molecule functionalized and the amount bound to the surface of the CNTs [6]. The results presented here suggest that the 46 kDa protein, used for this study, could lead to toxicity and this effect was potentiated by binding to CNTs as this system increases biodisponibility. Therefore, the amount of this functionalized protein may be another determinant of cellular toxicity. This is because in the P46P-CNTs, where functionalization did not reach 5% wt, no toxic effects were observed compared to P46S-CNTs.

### 3.3. Apoptosis Assays

**Figure 4:**
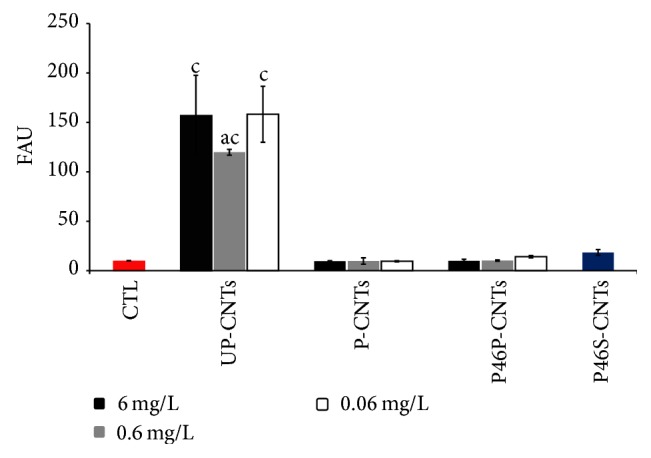
Apoptosis determination of MOs exposed to different CNTs at 24 h. Each bar represents mean ± SD of one experiment done in triplicate (*n* = 3). a, *P* < 0.01 denotes significant differences between mean values measured in the indicated group compared to the control without stimulus (CTL); b, *P* < 0.01 denotes significant differences between mean values for CNTs at different concentrations; c, *P* < 0.01 denotes significant differences between mean values for a particular concentration among different CNTs.

Cell apoptosis was determined by measuring caspase-3 activity in lysates of MOs exposed to the different CNTs. Caspase-3 is an enzyme that is activated in both intrinsic and extrinsic pathways of apoptosis; thus the increase in its activity reflects the induction of apoptosis in cells. Caspase-3 activity in MOs exposed to different CNTs are displayed in Figure 4; exposure of MOs to UP-CNT induced a significant increase in Caspase-3 activity at all concentrations tested compared to the control without stimulus. In contrast, the exposure of MOs to P-CNTs, P46P-CNTs, or P46S-CNTs resulted in no difference of caspase-3 activity compared with the control. These results agree with those obtained in the viability assays where MOs exposed to UP-CNTS had a decrease in the number of viable cells compared to the other treatments. These results are similar to those previously reported [4, 21]; some authors suggest that apoptosis induction is a process mediated by oxidative stress [28]. Moreover, MOs exposed to P46S-CNTs, which resulted in a significant decrease in cell viability, did not display an increase in caspase-3 activity. This result suggests that the toxic effect of P46S-CNTs could be related to a different mechanism other than cellular apoptosis. Although there are several proteins reported in* Entamoeba histolytica* corresponding to the molecular weight of 46 kDa, the identity as well as function of the functionalized specific protein is not known.

### 3.4. Morphologic Analysis

**Figure 5:**
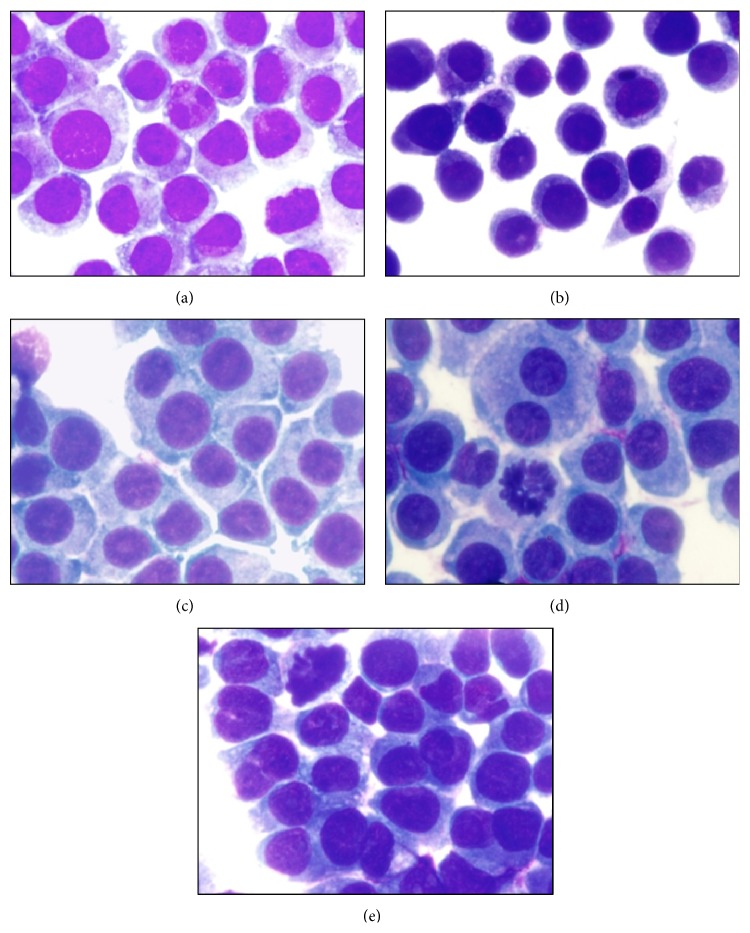
Photomicrographs of morphological changes detected in MOs exposed to CNTs. Photomicrographs of MOs stained with eosin and yellowish-methylene azure blue (original magnification 40x) as follows: (a) control without stimulus; (b) exposed to UP-CNTs; (c) exposed to P-CNTs; (d) exposed to P46P-CNTs; and (e) exposed to P46S-CNTs.

Lastly, morphology alterations were detected. MOs were exposed to different CNTs for 24 h and stained using a quick stain; results are shown in Figure 5. As is depicted in Figure 5(a), control MOs without stimulus displayed normal morphology; cells were predominantly uninuclear and had a well-defined monolayer. MOs exposed to P-CNTs or P46P-CNTs (Figures 5(c) and 5(d), resp.) also displayed normal morphology, whereas MOs exposed to UP-CNT (Figure 5(b)) had irregularities in their cytoplasm, the nuclei presented microcytic and condensed, and a loss of cell monolayer confluence was observed. These alterations are characteristic of apoptosis and are consistent with the results of viability and the caspase-3 activity. Similar results were reported in previous studies in different cell lines [4, 21]. On the other hand, MOs exposed to P46S-CNTs (Figure 5(e)) had slightly deformed cytoplasm and a slight decrease in cell monolayer confluence; however no changes in the nucleus were observed. These results confirm those obtained in the caspase-3 activity assays, in which no induction of apoptosis was detected. Slight changes observed in the morphology of the MOs demonstrated that apoptosis was not effectively induced in the cells; further studies are needed to elucidate the cytotoxic mechanism related to these nanoparticles.

## 4. Conclusions

The results presented in this paper permit us to conclude that the toxicity of CNTs is affected by not only its size and dispersion in aqueous solution but also the degree of functionalization, which can influence the dispersion and hence toxicity. Moreover, the nature of the molecule added to the CNTs may influence its toxic potential, as occurs with P46S-CNTs. Therefore, we can conclude that despite having evidence that* f-*CNTs produced no toxic effect, prior cytotoxic studies to evaluate the performance of these new nanoparticles should be required because this system increases the biodistribution of the adhered substance.
